# Impacts of Nonsynonymous Single Nucleotide Polymorphisms of Adiponectin Receptor 1 Gene on Corresponding Protein Stability: A Computational Approach

**DOI:** 10.1155/2016/9142190

**Published:** 2016-05-15

**Authors:** Md. Abu Saleh, Md. Solayman, Sudip Paul, Moumoni Saha, Md. Ibrahim Khalil, Siew Hua Gan

**Affiliations:** ^1^Department of Biochemistry and Molecular Biology, Jahangirnagar University, Savar, Dhaka 1342, Bangladesh; ^2^Human Genome Centre, School of Medical Sciences, Universiti Sains Malaysia, 16150 Kubang Kerian, Kelantan, Malaysia

## Abstract

Despite the reported association of adiponectin receptor 1 (*ADIPOR1)* gene mutations with vulnerability to several human metabolic diseases, there is lack of computational analysis on the functional and structural impacts of single nucleotide polymorphisms (SNPs) of the human* ADIPOR1* at protein level. Therefore, sequence- and structure-based computational tools were employed in this study to functionally and structurally characterize the coding nsSNPs of* ADIPOR1* gene listed in the dbSNP database. Our* in silico* analysis by SIFT, nsSNPAnalyzer, PolyPhen-2, Fathmm, I-Mutant 2.0, SNPs&GO, PhD-SNP, PANTHER, and SNPeffect tools identified the nsSNPs with distorting functional impacts, namely, rs765425383 (A348G), rs752071352 (H341Y), rs759555652 (R324L), rs200326086 (L224F), and rs766267373 (L143P) from 74 nsSNPs of* ADIPOR1* gene. Finally the aforementioned five deleterious nsSNPs were introduced using Swiss-PDB Viewer package within the X-ray crystal structure of ADIPOR1 protein, and changes in free energy for these mutations were computed. Although increased free energy was observed for all the mutants, the nsSNP H341Y caused the highest energy increase amongst all. RMSD and TM scores predicted that mutants were structurally similar to wild type protein. Our analyses suggested that the aforementioned variants especially H341Y could directly or indirectly destabilize the amino acid interactions and hydrogen bonding networks of ADIPOR1.

## 1. Introduction

In recent years, the number of obese individuals has been dramatically increased throughout the world which leads to the acceleration of obesity related health problems [1, 2]. Decreased insulin sensitivity, the most common arena of obesity, predisposes the affected persons to a variety of pathological abnormalities including type 2 diabetes, hypertension, and cardiovascular diseases [3–5]. The concomitance of these diseases has been considered as metabolic syndrome. In multiple studies, it has been reported that genetic variations in the adiponectin gene are associated with these types of diseases [6]. Adiponectin is an adipokine or adipocytokine specially secreted by adipocytes [7] and placenta during pregnancy [8] that circulates at relatively high (2–20 mg/mL) concentrations in the blood stream. Biologically active adiponectin hormone is a collagen-like circulating protein which acts as a principle antidiabetic and antiatherogenic adipokine [9–12]. Reduced adiponectin level in plasma has been observed in obesity, insulin resistance, and type 2 diabetes [9–12]. Adiponectin exerts its insulin sensitizing effects by increasing fatty-acid oxidation via activation of AMP-activated protein kinase (AMPK) peroxisome proliferator-activated receptor-alpha (PPAR-*α*) [13, 14]. Therefore, adiponectin is anticipated to be a novel therapeutic target for diabetes and the metabolic syndrome.

To employ proper functions, adiponectin binds to a number of receptors. Different studies have identified two receptors named adiponectin receptor-1 (ADIPOR1) and adiponectin receptor-2 (ADIPOR2) (those are homologous to G protein-coupled receptors) as well as one receptor similar to the cadherin family [15, 16]. In human beings,* ADIPOR1* and* ADIPOR2* genes are located at chromosomal locations 1p36.13-q41 and 12p13.31, respectively [17]. The expression of* ADIPOR1* gene is found principally in skeletal muscles but may be presented ubiquitously also, while the expression of* ADIPOR2* is the most abundant in liver [17]. Among these receptors, ADIPOR1 plays crucial roles in regulation of energy homeostasis as well as glucose and lipid metabolism [18]. According to several studies conducted, single nucleotide polymorphisms (SNPs) in* ADIPOR1* gene can hamper the physiological functions exerted by the ADIPOR1 protein. A recent comprehensive investigation on a European-Australian population has established the association of genetic variation in adiponectin receptors with type 2 diabetes [19, 20]. Moreover, some SNPs of* ADIPOR1* gene have been found to exert significant effects on the risk of prostate cancer (rs12733285) [21], insulin resistance (rs1342387) [22], and even liver fat deposition (−1927 T/C) [23]. Although there are several* in vivo* studies describing the association of SNPs in the* ADIPOR1* gene with metabolic disorders [24, 25], computational analysis has not yet been undertaken on the functional and structural consequences of nsSNPs in this gene.

In current years, computational tools are being widely used to characterize the impacts of deleterious nsSNPs in candidate genes by utilizing the information obtained from physicochemical properties of polypeptides [26, 27], conserved sequences across the species [28], and their structural attributes [29]. With the help of computational algorithms, several* in silico* studies have effectively filtrated functional SNPs out of large pool of diseases sensitive SNPs of* BRCA1, ATM* [30], and* PON1* [31] genes based on their functional consequences and structural stabilities. In spite of the availability of undoubted data referring the extensive involvement of* ADIPOR1* gene mutations in human diseases, the computational analysis of nsSNPs is still unveiled.

In this study, the clinical variants of* ADIPOR1* were collected for* in silico* analysis. By utilizing these data, we employed different publicly available bioinformatics tools and databases for a comprehensive analysis of nsSNPs in* ADIPOR1* gene. We also calculated the free energy changes for mutants and wild type ADIPOR1 protein in order to evaluate their stability. This study might be helpful for further investigation in order to discover new therapeutic drugs related to adiponectin receptor 1 associated diseases.

## 2. Methods and Materials

### 2.1. SNP Data Mining

The data on the human* ADIPOR1* gene were obtained from web-based data sources such as Online Mendelian Inheritance in Man (OMIM; http://www.ncbi.nlm.nih.gov/omim/) and the National Center for Biological Information (http://www.ncbi.nlm.nih.gov/). The information about SNPs of* ADIPOR1* gene of* Homo sapiens* was collected from the dbSNP-NCBI (http://www.ncbi.nlm.nih.gov/SNP/) [32] for further computational analysis. The protein sequence of* ADIPOR1* gene was obtained from UniProtKB database (http://www.uniprot.org/uniprot/).

### 2.2. Analysis of the Functional Consequence of nsSNPs by Sorting Intolerant from Tolerant (SIFT)

SIFT (http://sift.jcvi.org/) predicts the deleterious and tolerated SNPs in order to characterize the consequences of amino acid substitutions on phenotypic and functional changes of protein molecules. By using sequence homology based method, SIFT assumes that significant positions in a protein sequence have been conserved throughout evolution and, therefore, substitutions at these positions may affect protein function. The identification numbers (rsIDs) of each nsSNP of* ADIPOR1* gene were submitted as an input to SIFT server for homology searching. SIFT calculates the SIFT score or tolerance index (TI) score for each nsSNP. The SIFT value ≤ 0.05 indicates the deleterious effect of nonsynonymous variants on protein function [33].

### 2.3. Investigation of Functional Impacts of nsSNPs by nsSNPAnalyzer

nsSNPAnalyzer (http://snpanalyzer.uthsc.edu/) server was used to predict whether a nsSNP of ADIPOR1 protein affects its phenotypic effect. The input options for nsSNPAnalyzer are protein sequences in FASTA format and detailed information on amino acid substitutions. This server usually uses information contained in the multiple sequence alignment and the 3D structure in order to make a prediction. The prediction of this tool is based on a machine learning method known as Random Forest. The results of this server depict whether an nsSNP is associated with disease or neutral [34].

### 2.4. Analysis of the Functional Impacts of nsSNPs by Screening for Nonacceptable Polymorphisms (SNAP2)

To find the functional effects of nsSNP, SNAP2 (https://rostlab.org/services/snap2web/) server was used. The prediction done by SNAP2 is based on a learning device method known as neural network. In order to make a prediction, SNAP2 utilizes the information of automatically created multiple sequence alignment and also some structural features such as predicted secondary structure and solvent accessibility. FASTA format of protein sequences is only the input option for SNAP2. The output of this server consists of prediction (Effect or neutral), score (ranges from −100 strong neutral prediction to +100 strong effect prediction), and expected accuracy [35].

### 2.5. Characterization of Functional Consequence of nsSNPs by PolyPhen-2

PolyPhen-2 (http://genetics.bwh.harvard.edu/pph2/) is an advanced version of the PolyPhen tool that was used to find out the possible effect of an amino acid substitution on the structure and function of ADIPOR1 protein. UniProtKB accession number/FASTA sequence and details of amino acid substitutions are required for the input options of PolyPhen-2 server. This tool calculates Naïve Bayes posterior probability that this mutation is damaging and reports estimation of corresponding false positive and true positive rate. A mutation is estimated qualitatively as probably damaging (probabilistic score >0.85), possibly damaging (probabilistic score >0.15), and benign (remaining) with specificity and sensitivity values [36].

### 2.6. Prediction of Disease Related nsSNPs by SNPs&GO

SNPs&GO (http://snps.biofold.org/snps-and-go/snps-and-go.html) is a support vector machine (SVM) based classifier [37]. This server accurately predicts the mutation related to disease from protein sequence. The probability score greater than 0.5 indicates that the disease related effect is caused by nsSNPs on the function of parent protein. The whole protein sequence in FASTA format is the input for this server. The server also provides the output display for additional two servers such as PHD-SNP [38] and PANTHER [39] algorithms.

### 2.7. Functional Analysis of nsSNP through Hidden Markov Models (Fathmm)

Fathmm (http://fathmm.biocompute.org.uk/inherited.html) not only predicts the potentially deleterious nature of protein variants but also the skill of annotating the molecular and phenotypic consequences of these mutations [40]. This server is composed of two algorithms: sequence/conservation based (unweighted) and other combined sequence conservation with pathogenicity weights (weighted). In this study, we used weighted algorithm because this algorithm is capable of adjusting conservation-based predictions to account for the tolerance of related sequences to mutations.

### 2.8. Investigation of the Molecular Phenotypic Effects of nsSNPs by SNPeffect

The SNPeffect database 4.0 (http://snpeffect.switchlab.org/) utilizes sequence- and structure-based bioinformatics tools in order to make prediction of molecular phenotypic impacts of nsSNP on* ADIPOR1* gene. This server mainly integrates three different tools such as TANGO, WALTZ, and LIMBO and also uses FoldX server to find out a decision whether the mutation is stabilizing or destabilizing the structure of native proteins. TANGO algorithm identifies the aggregation prone regions in a protein sequence by calculating the hydrophobicity and beta-sheet forming propensity. WALTZ algorithm predicts amyloid forming regions in protein sequences with accuracy and specificity, while LIMBO algorithm predicts a chaperone binding site for the Hsp70 chaperones. The input options are usually composed of FASTA sequence/PDB ID/PDB file/UniProt ID and details of nsSNP [41].

### 2.9. Prediction of Protein Stability Changes upon nsSNPs by I-Mutant 2.0

I-Mutant 2.0 (http://folding.biofold.org/i-mutant/i-mutant2.0.html) is a support vector machine- (SVM-) based tool which was used to predict the protein stability changes upon nsSNPs. In this study, sequence of protein, temperature (25°C), pH (7), and details of nsSNPs were used as input parameters to this server. The output is a free energy change value (ΔΔG) of protein after and before mutation. Positive ΔΔG value concludes that the protein being mutated is of higher stability and vice versa is also true [42].

### 2.10. Identification of Functional Regions in Proteins by ConSurf

ConSurf (http://consurf.tau.ac.il/) is a web-based tool that automatically analyzes evolutionary conservation of amino acid substitutions in protein by using an empirical Bayesian inference. This server is composed of combining two self-governing servers (ConSeq and ConSurf). After providing the FASTA sequence of ADIPOR1 protein to ConSurf tool, the conserved regions were predicted with conservation grades color-coded onto its surface that can finally be pictured online using the Protein Explorer engine [43].

### 2.11. Analysis of Impacts of nsSNPs on Surface and Solvent Accessibility of Protein by NetSurfP

The active site of a protein in its three-dimensional conformation can be traced by surface and solvent accessibility region of amino acids of that protein. The FASTA sequence of ADIPOR1 protein was submitted to NetSurfP (http://www.cbs.dtu.dk/services/NetSurfP/) server in order to predict its secondary structure, surface, and solvent accessibility of amino acids [44]. The output of this server provides 3 subclasses defined for solvent accessibility of amino acids: low accessibility (buried), moderate accessibility (partially buried), and high accessibility (exposed).

### 2.12. Modeling the Molecular Effects of nsSNPs on Protein Structure and Evaluating Their Difference of RMSD Value and TM Score

Structural analysis was done in order to explore the structural deviations and stability differences between native and mutant forms of ADIPOR1 proteins. The crystal structure of ADIPOR1 protein available in Protein Data Bank (PDB) [45] has an ID 3WXV. The ADIPOR1 protein contains 375 amino acids from which 287 amino acids have been resolved in crystal structure with a resolution of 2.90 Å [46]. The Swiss-PDB viewer [47] was utilized in order to carry out amino acid substitutions, followed by the energy minimization of the modeled 3D structure of variants using a version of the GROMOS 43B1 force field in GROMOS96 software package embraced in the Swiss-PDB viewer. TM-Align was used to calculate the TM scores and root mean square deviations (RMSDs) [48].

### 2.13. Identification of Ligand Binding Sites on Unbound Protein Structure by FTSite

Detection of ligand binding sites on unbound proteins is essential to elucidate the protein structure-function relation and for protein engineering. FTSite (http://ftsite.bu.edu/) predicts ligands or small molecule binding sites of proteins based on experimental evidence with 94% accuracy [49]. The input options of this server generally consist of job name, Protein Data Bank ID (PDB ID) or file, and also PDB chain ID if proteins contain multiple subunits.

### 2.14. Investigation of Protein-Protein Interactions

Protein-protein interaction networks are important to investigate the functions of the interactions of a particular protein with other proteins at cellular level. Online database resource Search Tool for the Retrieval of Interacting Genes (STRING) was applied to identify the interactions of ADIPOR1 protein with other corresponding proteins [50]. This server provided a unique coverage and ease of access to both experimental and predicted interaction information of ADIPOR1. In this study, we operated KEGG (http://www.genome.jp/kegg/) PATHWAY and LIGAND to make prediction of the functional networking of ADIPOR1 protein.

## 3. Results and Discussion

### 3.1. Retrieval of SNPs

The dbSNP-NCBI database was searched for retrieving the SNPs in the human* ADIPOR1* gene (Gene ID: 51094). A total of 138 SNPs were found in the exonic region, among them 62 (44.93%) were synonymous, 74 (53.62%) nonsynonymous and missense, 1 (0.72%) nonsynonymous and nonsense, and 1 (0.72%) frame-shift mutations. However, only nonsynonymous SNPs were selected from coding region for this computational analysis.

### 3.2. Detection of Functional nsSNPs in Exonic Regions

The searching of functionally significant nsSNPs was done by predicting those which substitute the amino acids that are critical for* ADIPOR1* gene function. This computational study was accomplished and authenticated using different* in silico* tools, namely, SIFT, nsSNPAnalyzer, SNAP2, PolyPhen-2, SNPs&GO, Fathmm, SNPeffect, and I-Mutant 2.0.

#### 3.2.1. Analysis of Phenotypic Impacts by SIFT

SIFT tools filtrated that a total of 13 variants (17.568%) were damaging (score of 0.00–0.04) and the remaining 61 variants (82.432%) became tolerated (score of 0.08–0.55). It was noted that, among 13 variants, 2 nsSNPs (rs765487840, rs775780092) were predicted as damaging with low confidence. Therefore, SIFT suggested that these 11 nsSNPs might disrupt both the protein function and structure. The detailed results are provided in supporting information (see Table S1 of the Supplementary Material available online at http://dx.doi.org/10.1155/2016/9142190).

#### 3.2.2. Functionally Significant nsSNPs by nsSNPAnalyzer and SNAP2

The results obtained from the nsSNPAnalyzer (Table S2) predicted that a total of 27 nsSNPs (36.486%) might be disease causal. In contrast, 47 nsSNPs (63.513%) have no effect on protein function and, hence, are considered as neutral. In addition, the results from SNAP2 server (Table S2) indicated 19 variants (25.675%) as significant and the remaining nsSNPs (74.324%) as neutral. Among the three computational tools, the highest number of significant nsSNP (27 variants) was detected by the nsSNPAnalyzer. The results obtained from SIFT, nsSNPAnalyzer, and SNAP2 concluded that the 7 nsSNPs with rsIDs of rs764078304, rs765425383, rs752071352, rs759555652, rs764912508, rs200326086, and rs766267373 are found as significant among three servers and thereby the result has one step refined and validated (Table 1).

#### 3.2.3. Simulation of Functional Consequences by PolyPhen-2

The results (Table S3) obtained from PolyPhen-2 server indicated that 12 (16.216%) out of 74 nsSNPs were predicted as probably damaging (score of 0.96–1.00; more confident prediction) and 12 (16.216%; less confident prediction) nsSNPs were ranked as possibly damaging (score of 0.531–0.874) as well. Meanwhile, 50 (67.567%) nsSNPs were also classified as benign (score of 0.411–0.000). The classification of SNPs on the basis of PolyPhen-2 scores permits us to assess the potential quantitative effect of SNPs on wild type protein. Moreover, 7 nsSNPs (rs765425383, rs752071352, rs759555652, rs200326086, rs772408783, rs766267373, and rs749789403), predicted as damaging by SIFT, are also found as damaging using PolyPhen-2. This result gives clear indication that there is a strong correlation exists between evolutionary based approaches SIFT and the structural based approach PolyPhen-2 tools.

#### 3.2.4. Functional Characterization by PhD-SNP, PANTHER, SNPs&GO, and Fathmm

We performed PhD-SNP, PANTHER, SNPs&GO, and Fathmm analyses of human* ADIPOR1* nsSNPs in order to add another layer of refinement in nsSNPs characterization. The predicted results by these servers are shown in Tables S4 and S5.

The predictions gained form PhD-SNP server offer the fact that 29 nsSNPs cause disease with probability score greater than 0.5 and the remaining nsSNPs are marked as neutral. The number of disease causing variants has been decreased in case of the prediction of PANTHER and SNPs&GO tools. The disease causing variants predicted by PANTHER and SNPs&GO are 15 and 13 nsSNPs, respectively. The results from PANTHER server showed that 17 nsSNPs remain unclassified.

From Fathmm, the nsSNPs in amino acids positions 4 to 122 in human ADIPOR1 protein are found to be damaging with score of −3.97 to −4.20.

The efficacy of functional SNP prediction can be increased more reliably by integrating the results of SVM based approaches. By combining the predictions of SIFT, nsSNPAnalyzer, SNAP2, PolyPhen-2, PhD-SNP, PANTHER, SNPs&GO, and Fathmm, five nsSNPs (A348G, H341Y, R324L, L224F, and L143P) are found to br more deleterious and disease associated (Tables 1 and 2).

#### 3.2.5. Functional Investigation by SNPeffect

Biological macromolecules including proteins undergo self-assembly into functional complex in a tightly regulated manner to conduct the defined function [51]. Failure of correct aggregation of proteins may result in some conditions including type 2 diabetes, Alzheimer's disease, and other neurological diseases [52]. The results from TANGO investigation presented that only two variants, namely, A348G (dTANGO score is −39.32) and R324L (dTANGO score is −1.01), were found to be not affected among 5 selected variants in the aggregation prone regions of ADIPOR1 protein. In addition, the aggregation tendency of the other two variants, H341Y (dTANGO score is 222.09) and L224F (dTANGO score is 51.62), was increased and only one variant (L143P) with dTANGO score of −230.61 was decreased. On the other hand, WALTZ analysis screened that H341Y mutant (dWALTZ score −241.53) was found to be decreased to protein amyloid forming propensity and the rest of mutants were not affected. LIMBO prediction revealed that no variants were detected to modify the chaperone binding sites for Hsp70 chaperones. In this study, we analyzed the variants by SNPeffect tools at 90% homology searching of protein structures. SNPeffect could not find any reliable structural information for protein to carry out a FoldX stability analysis. Detailed results for selected 5 variants are supplied in Table 3.

#### 3.2.6. Protein Stability Changes Found by I-Mutant 2.0

The prediction of stability changes of selected 5 nsSNPs by I-Mutant 2.0 is given in Table 3. The results are predicted to be either increase or decrease of the free energy change upon amino acid substitutions. Four out of five selective mutants were found to be decreased in protein stability and the remaining one mutant (H341Y) was predicted as increased in protein stability with reliability index (RI) 3.

#### 3.2.7. Visualization of Evolutionary Conserved Amino Acid Residues by ConSurf

ConSurf server is able to discriminate appropriately between the conservation caused by a short evolutionary time and genuine sequence conservation using Empirical Bayesian method. Our findings indicated that human* ADIPOR1* is highly conserved (Figure 2). The sequence alignment from different species revealed that residues A348 and H341 were located in highly conserved regions and predicted to cause structural and functional impacts, respectively, on ADIPOR1 protein. On the other hand, the residues L224 and L143 had average conserved scores and the remaining one residue (R324) was located in conserved region of the protein.

### 3.3. Structural Analysis of Mutant Structures

The five predicted deleterious and disease causing variants were mapped to the PDB ID 3WXV native structure and substitution of amino acid residues was carried out using Swiss-PDB Viewer individually in order to generate five mutant modeled structures. After that, we calculated the total energy before and after energy minimization for both mutant model and wild type structures (Table 5). The values of total energy for five mutant modeled structures exhibit deviation from native structure considered before and after energy minimization. Five modeled structures (A348G, H341Y, R324L, L224F, and L143P) revealed an increase in energy (less favorable change) after energy minimization in comparing native structure. Among five screened mutations, H341Y showed the highest increase in energy which may be explained by the energetically unfavorable substitution of His to Tyr amino acids. The zinc-binding domain is found in the intracellular layer of the membrane and zinc ion is coordinated by three His residues, His191, His337, and His341, of ADIPOR1 protein. In H341Y variants, His is replaced by Tyr. Due to the presence of aromatic amino acid Tyr in 341 position of ADIPOR1 protein there may be a good chance to disrupt zinc coordination. Adiponectin stimulated AMPK phosphorylation and UCP2 upregulation are mediated by zinc-binding domain [46].

The results from TM score are delivered in Table 6. TM score was utilized in order to evaluate the topological similarity of two protein structures and RMSD measured the average distance between the backbones of two superimposed proteins [53]. The TM score for five variants reveals that structurally there are no differences between native and mutant modeled structures. It might be concluded that mutants and wild type structures are matched perfectly. We also considered another parameter (RMSD) in order to predict the structural similarity between native and mutant structures of ADIPOR1 protein. The higher is the RMSD value, the more is the deviation between the two structures which in turn fluctuates their functional activities. It can be seen from Table 6 that the RMSD values between the native structure and the mutant modeled structures are all similar. By considering the above two values of TM score and RMSD, it could be suggested that these mutations do not bring a significant alteration in the mutant structures with regard to the native protein structure.

**Figure 1:**
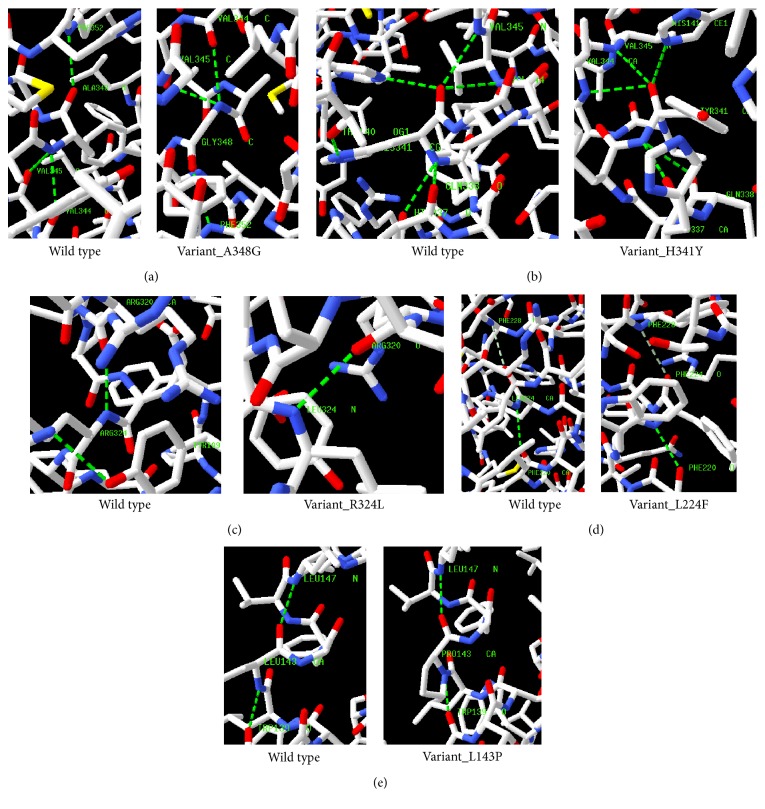
H-bond (green discontinuous line) and weak hydrogen bond (dark white discontinuous line) of wild type and mutant analogues with the adjacent amino acids residues. (a) At 348 position, three hydrogen bonds (H-bond) are observed with Val344, Val345, and Phe352 in both wild type (Ala348) and mutant (Gly348) structures. (b) His341 is visualized with six H-bonding interactions for Thr140, His141, His337, Gln338, Val344, and Val34, and one H-bond is abolished due to the replacement of mutant Tyr34 at the same position. (c) At 324 position, two H-bonds are observed with Tyr109 and Arg320 in native (Arg324) structure, but only one H-bond is found with Arg320 in mutant (Leu324) structure at the same position. (d) Phe228 is examined with single weak H-bonding in both native (Leu224) and substituted (Phe224) structures. In addition, single H-bond is also pictured with Phe220 in both structures. (e) Trp139 and Leu147 participated in forming two H-bonds at same position (143) in both mutant and wild type structures.

**Figure 2:**
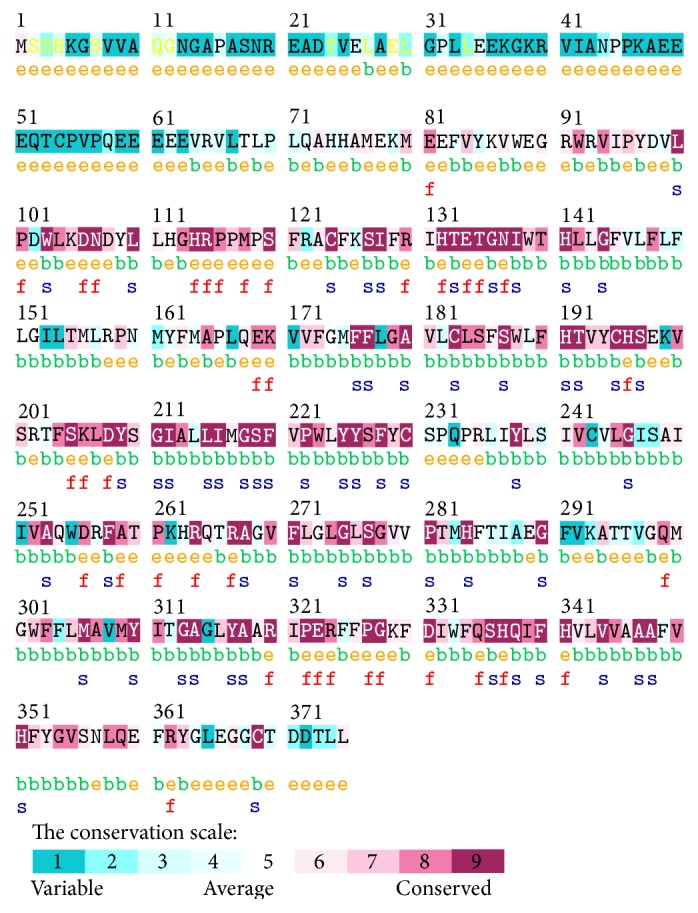
Unique and conserved amino acids in ADIPOR1 protein were predicted by ConSurf. Amino acids were ordered based on a conservation scale of 1–9 and highlighted as follows: blue residues (1–4) are variable, white residues (5) are average, and purple residues (6–9) are conserved. (e) Exposed residues are colored via an orange letter. (b) Buried residues are marked via a green letter. (f) Putative functional highly conserved and exposed residues are revealed with a red letter. (s) Predicted structural residues which are highly conserved and buried are indicated via blue letter.

Nonbonding interactions such as H-bond has significant role in stabilizing the secondary structure of proteins [54]. Therefore, we have utilized the Swiss-PDB Viewer to visualize the hydrogen bonding pattern of five selected substituted amino acids with their surrounding amino acid residues in mutant proteins with regard to wild type (Figure 1). The hydrogen bonding pattern of variants A348G, L224F, and L143P has remained similar in comparison with wild type structure (PDB ID 3WXV). In variant H341Y, His341 indicates six hydrogen bonding interactions with Thr140, His141, Val344, Val345, His337, and Gln338, whereas mutant aromatic Tyr341 indicates five hydrogen bonding interactions. This has occurred due to the differences in the charge density and hydrophobicity between wild type and mutant residues. In variant R324L, one H-bond disappeared due to the substitution of Arg324 by Leu324. Additionally, A348G, H341Y, R324L, L224F, and L143P variants were analyzed for solvent accessibility and stability and significant changes in both parameters were seen for all five variants (Table 4).

### 3.4. Analysis of Ligand Binding Sites and Protein-Protein Interactions

**Figure 3:**
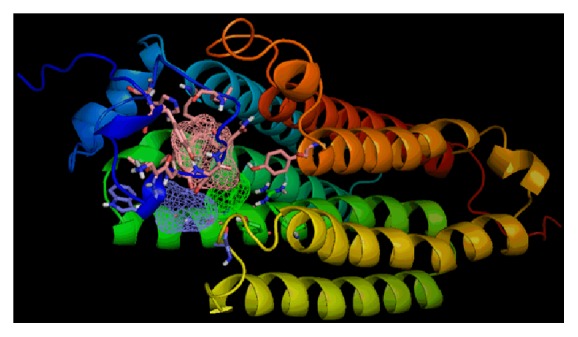
Binding of ligands in ADIPOR1 proteins ligand binding pocket 1-3 predicted by FTSite. No mutants were observed in binding site 1-3.

FTSite identifies 3 ligand binding sites on ADIPOR1 protein (Figure 3). The amino acids found in these 3 sites of ADIPOR1 protein are given in Table 7. By the results of FTSite, it is observed that our 5 selected variants are not involved among these sites.

**Figure 4:**
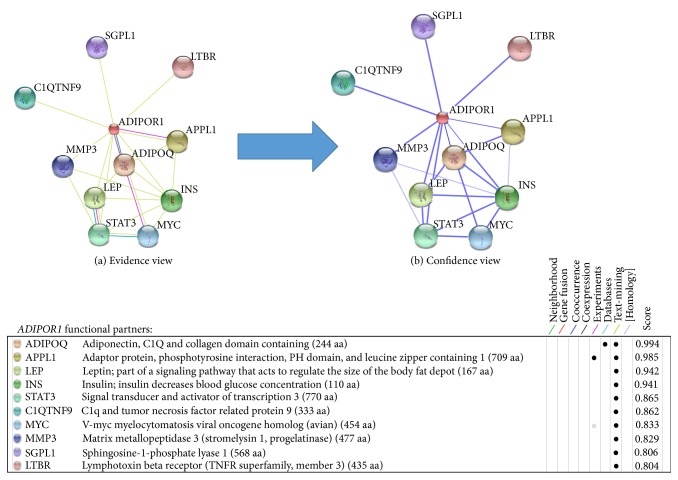
ADIPOR1 protein-protein interactions with 10 partners. One color is specified for each type of evidence in the predicted functional links (edges) among eight colored lines. (a) Only* APPL1* with score of 0.985 is observed for interaction with* ADIPOR1* by experimental and text-mining basis. From text-mining data,* ADIPOR1* interactions are detected for* ADIPOQ, APPL1, LEP, INS, STAT3, C1QTNF9, MYC, MMP3, SGPL1,* and* LTBR* proteins with 0.994, 0.985, 0.942, 0.941, 0.865, 0.862, 0.833, 0.828, 0.806, and 0.804 scores, respectively. (b) Strong association pattern (thick blue lines) of* ADIPOR1* is predicted for* ADIPOQ, APPL1, LEP,* and* INS* partners with high confidence. The remaining partners have weak association and shown in the form of thin blue lines.

STRING database predicted the functional interaction pattern of ADIPOR1 protein to other proteins in a cell. Strong functional associations of ADIPOR1 protein have been observed with* ADIPOQ, APPL1, LEP,* and* INS* partners (Figure 4). Besides, weak interactions with less confidence have been observed for* STAT3, C1QTNF9, MYC, MMP3, SGPL1,* and* LTBR* proteins.

The associations between polymorphisms of* ADIPOR1* gene (such as rs12733285 and rs1342387) and metabolic diseases such as diabetes, obesity, and insulin resistance have been reported [19, 20, 22, 23]. However, no such study has established the association between damaging nsSNPs (rs765425383, A348G; rs752071352, H341Y; rs759555652, R324L; rs200326086, L224F; and rs766267373, L143P) and diseases. Hence, the confirmation of these nsSNPs in any disease is required to complement the existing limited body of knowledge. The combination of the analysis of human genetic variations of the* ADIPOR1* gene together with the computational method to predict their possible functional impact may help in the analysis of* ADIPOR1* gene variants and establish their effects on protein functional characteristics. Specifically, this approach permits the estimation of the probability of amino acid changes which can be detrimental for ADIPOR1 protein functions.

## 4. Conclusion

In this comprehensive computational study, we have identified five deleterious mutations (A348G, H341Y, R324L, L224F, and L143P) among the coding region of* ADIPOR1* gene with the help of different bioinformatics tools. The variants were predicted to be similar to wild type ADIPOR1 protein structurally. However, decreased stability of mutant proteins has been observed with classical molecular dynamics study compared to wild type. Among the potential five nsSNPs, H341Y mutant has been found to cause considerable changes in amyloid forming propensity and aggregation tendency of ADIPOR1 protein. Additionally, there might be chance to disrupt the zinc coordination domain which is responsible for adiponectin stimulated MPK phosphorylation and UCP2 upregulation. The deleterious mutations of* ADIPOR1* should be further investigated to establish their roles in the pathogenesis of related diseases.

## Supplementary Material

Table S1 to S5 depict results of nonsynonymous SNPs of the human *ADIPOR1* gene analyzed by SIFT, SNAP2, nsSNPAnalyzer, PolyPhen-2, PhD-SNP, PANTHER, SNPs&GO, and Fathmm.

## Figures and Tables

**Table 1:** Refined SNPs obtained from SIFT, SNAP2, and nsSNPAnalyzer based classifications system.

rsIDs	Amino acid change	SIFT		SNAP2		nsSNPAnalyzer
		Score	Prediction	Score	Prediction	Prediction
rs764078304	*G367R*	*0.04*	*Damaging*	*72*	*Effect*	*Disease*
rs139371614	G364S	0.79	Tolerated	−74	Neutral	Disease
rs765425383	*A348G*	*0*	*Damaging*	*34*	*Effect*	*Disease*
rs752071352	*H341Y*	*0*	*Damaging*	*80*	*Effect*	*Disease*
rs759555652	*R324L*	*0.02*	*Damaging*	*56*	*Effect*	*Disease*
rs778848411	V279G	0.02	Damaging	36	Effect	Neutral
rs759593783	G275A	0.24	Tolerated	45	Effect	Disease
rs764912508	*R264W*	*0.02*	*Damaging*	*49*	*Effect*	*Disease*
rs369530077	I251N	0.01	Damaging	19	Effect	Neutral
rs200326086	*L224F*	*0.01*	*Damaging*	*2*	*Effect*	*Disease*
rs772408783	L215V	0.02	Damaging	1	Effect	Neutral
rs756988796	R202W	0.08	Tolerated	−35	Neutral	Disease
rs772165061	V200L	0.23	Tolerated	8	Effect	Neutral
rs760115326	F173L	0.1	Tolerated	48	Effect	Disease
rs770463342	K170N	0.09	Tolerated	25	Effect	Neutral
rs767286210	L149F	0.32	Tolerated	−50	Neutral	Disease
rs780018580	F145L	0.75	Tolerated	−45	Neutral	Disease
rs766267373	*L143P*	*0.01*	*Damaging*	*75*	*Effect*	*Disease*
rs764226232	R122W	0.17	Tolerated	−36	Neutral	Disease
rs751626519	M118K	0.2	Tolerated	60	Effect	Disease
rs781585434	P116S	0.18	Tolerated	6	Effect	Neutral
rs749789403	D108N	0.02	Damaging	−10	Neutral	Neutral
rs141511034	P96L	0.33	Tolerated	−75	Neutral	Disease
rs769729230	E78K	0.15	Tolerated	31	Effect	Disease
rs751028180	G31E	1	Tolerated	−81	Neutral	Disease
rs149582032	A28T	0.61	Tolerated	−88	Neutral	Disease
rs780838176	E26Q	0.34	Tolerated	−89	Neutral	Disease
rs749145406	A15P	0.35	Tolerated	−34	Neutral	Disease
rs200868442	G14F	0.91	Tolerated	−4	Neutral	Disease
rs774465119	N13K	0.93	Tolerated	18	Effect	Disease
rs372656012	G12E	1	Tolerated	−63	Neutral	Disease
rs759643470	V9E	0.74	Tolerated	61	Effect	Disease
rs765487840	H4L	0.03	Damaging	−9	Neutral	Disease
rs775780092	H4Y	0.11	Damaging	−27	Neutral	Disease

**Table 2:** Refined SNPs obtained from PhD-SNP, PANTHER, SNPs&GO, Fathmm, and PolyPhen-2 based classification systems.

Amino acid change	PhD-SNP	PANTHER	SNPs&GO	Fathmm	PolyPhen-2
G367R	Disease	Neutral	Neutral	Tolerated	Benign
*A348G*	*Disease*	*Disease*	*Disease*	*Tolerated*	*Probably damaging*
*H341Y*	*Disease*	*Disease*	*Disease*	*Tolerated*	*Probably damaging*
*R324L*	*Disease*	*Disease*	*Disease*	*Tolerated*	*Probably damaging*
A307V	Disease	Neutral	Disease	Tolerated	Benign
T296R	Disease	Neutral	Neutral	Tolerated	Benign
V279G	Disease	Disease	Disease	Tolerated	Benign
G275A	Neutral	Disease	Disease	Tolerated	Probably damaging
V270M	Neutral	Disease	Neutral	Tolerated	Possibly damaging
R264W	Disease	Disease	Disease	Tolerated	Possibly damaging
I251N	Disease	Disease	Disease	Tolerated	Possibly damaging
S231P	Disease	Neutral	Neutral	Tolerated	Possibly damaging
*L224F*	*Disease*	*Neutral*	*Neutral*	*Tolerated*	*Probably damaging*
L251V	Disease	Neutral	Neutral	Tolerated	Probably damaging
R202W	Disease	Disease	Disease	Tolerated	Benign
F173L	Disease	Neutral	Neutral	Tolerated	Possibly damaging
K170N	Neutral	Disease	Neutral	Tolerated	Probably damaging
F145L	Disease	Neutral	Neutral	Tolerated	Benign
*L143P*	*Disease*	*Disease*	*Disease*	*Tolerated*	*Probably damaging*
R130C	Disease	Disease	Disease	Tolerated	Benign
R122W	Disease	Disease	Neutral	Damaging	Benign
M118K	Disease	Disease	Disease	Damaging	Benign
P116S	Neutral	Disease	Neutral	Tolerated	Probably damaging
D108N	Disease	Neutral	Neutral	Tolerated	Probably damaging
R91H	Disease	Neutral	Neutral	Tolerated	Benign
E89D	Disease	Neutral	Disease	Tolerated	Benign
E78K	Disease	Neutral	Neutral	Tolerated	Benign
P70S	Neutral	Neutral	Neutral	Tolerated	Probably damaging
R40P	Disease	Unknown	Neutral	Tolerated	Benign
G31E	Neutral	Unknown	Neutral	Tolerated	Probably damaging
A15P	Disease	Unknown	Neutral	Tolerated	Benign
G14F	Disease	Unknown	Neutral	Tolerated	Benign
N13K	Disease	Unknown	Neutral	Tolerated	Benign
G12E	Disease	Unknown	Neutral	Tolerated	Benign
V9E	Disease	Unknown	Neutral	Tolerated	Benign

**Table 3:** A list of selected variants for analyzing SNPeffect and I-Mutant tools.

Amino acid change	SNPeffect				I-Mutant	
	TANGO Aggregation tendency (dTANGO score)	WALTZ Amyloid propensity (dWALTZ score)	LIMBO Chaperone binding tendency (dLIMBO score)	FoldX Protein stability	Prediction	RI
A348G	Not affected	Not affected	Not affected	NP	Decrease	8
	(−39.32)	(46.87)	(0.00)			

H341Y	Increased	Decreased	Not affected	NP	Increase	3
	(222.09)	(−241.53)	(0.00)			

R324L	Not affected	Not affected	Not affected	NP	Decrease	7
	(−1.01)	(0.49)	(0.00)			

L224F	Increased	Not affected	Not affected	NP	Decrease	8
	(51.62)	(−46.88)	(0.00)			

L143P	Decreased	Not affected	Not affected	NP	Decrease	4
	(−230.61)	(2.58)	(0.00)			

**Table 4:** Surface accessibility of wild type and mutants of ADIPOR1 protein.

Amino acid change	Class assignment	Relative surface accessibility (RSA)	Absolute surface accessibility	*Z*-fit score for RSA prediction
A348*G*	Buried	0.094	10.403	−0.412
	*Buried*	*0.093*	*7.288*	*−0.405*

H341*Y*	Buried	0.070	12.769	0.848
	*Buried*	*0.063*	*13.463*	*0.712*

R324*L*	Exposed	0.34	77.860	−0.843
	*Exposed*	*0.352*	*64.470*	*−0.734*

L224*F*	Buried	0.029	5.273	−0.077
	*Buried*	*0.027*	*5.519*	*0.018*

L143*P*	Exposed	0.305	55.937	−0.824
	*Exposed*	*0.329*	*46.628*	*−0.942*

**Table 5:** Total energy of native and mutant ADIPOR1 structures before and after energy minimization.

Amino acid variants	Total energy before energy minimization (kj/mol)	Total energy after energy minimization (kj/mol)
Native	−6555.888	−11406.533
A348G	−6468.591	−11319.678
H341Y	86183.258	−10520.564
R324L	−6078.778	−11107.321
L224F	372036.875	−11199.636
L143P	179867.000	−11088.141

**Table 6:** RMSD value and TM score of mutant modeled structures of ADIPOR1 protein.

Variants	RMSD value	TM score
A348G	0.00	1.00
H341Y	0.00	1.00
R324L	0.00	1.00
L224F	0.00	1.00
L143P	0.00	1.00

**Table 7:** Residues at ligand binding sites of ADIPOR1 protein.

Site 1	Site 2	Site 3
TYR A 97	PHE A 190	TRP A 103
LEU A 104	SER A 205	LEU A 104
LYS A 105	ASP A 208	ASP A 106
ASP A 106	TYR A 209	PHE A 190
ASN A 107	ARG A 267	TYR A 194
LEU A 110	TYR A 317	SER A 201
HIS A 114		SER A 205
GLU A 134		ALA A 259
PHE A 190		
HIS A 191		
TYR A 194		
TYR A 317		
