# Supplementary material for: Impacts of Nonsynonymous Single Nucleotide Polymorphisms of Adiponectin Receptor 1 Gene on Corresponding Protein Stability: A Computational Approach
Source: Biomed Res Int. 2016 May 15;2016:9142190. doi: 10.1155/2016/9142190 (PMC4884590; doi:10.1155/2016/9142190)
Supplement: Supplementary file 1 — Table S1 to S5 depict results of nonsynonymous SNPs of the human ADIPOR1 gene analyzed by SIFT, SNAP2, nsSNPAnalyzer, PolyPhen-2, PhD-SNP, PANTHER, SNPs&GO, and Fathmm. [file 9142190.f1.docx]

| Table S1: List of non-synonymous SNPs of the human *ADIPOR1* gene analyzed by SIFT | | | | | |
| --- | --- | --- | --- | --- | --- |
|  | SNPs | | SIFT | | |
| rsID | dbSNP | Amino acid change | Prediction | Score | Median |
| rs764078304 | G/C | G367R | DAMAGING | 0.04­­ | 2 |
| rs139371614 | G/A | G364S | TOLERATED | 0.79 | 1.92 |
| rs766580369 | A/T | Y363F | TOLERATED | 0.74 | 1.89 |
| rs765425383 | C/G | A348G | DAMAGING | 0 | 1.91 |
| rs778182434 | G/A | V342I | TOLERATED | 0.35 | 1.85 |
| rs752071352 | C/T | H341Y | DAMAGING | 0 | 1.85 |
| rs759555652 | G/T | R324L | DAMAGING | 0.02 | 1.85 |
| rs780196089 | G/A | A319T | TOLERATED | 0.71 | 1.85 |
| rs763241138 | A/G | I311V | TOLERATED | 0.16 | 1.85 |
| rs12072660 | C/T | A307V | TOLERATED | .31 | 1.85 |
| rs374623170 | C/G | T296R | TOLERATED | 0.48 | 1.84 |
| rs757727437 | C/T | T295I | TOLERATED | 0.28 | 1.84 |
| rs781601173 | A/G | K293R | TOLERATED | 0.36 | 1.86 |
| rs749903174 | G/A | V292I | TOLERATED | 0.42 | 1.89 |
| rs755615066 | G/A | A288T | TOLERATED | 0.76 | 1.85 |
| rs779694619 | T/C | M283T | TOLERATED | 0.18 | 1.85 |
| rs778848411 | T/G | V279G | DAMAGING | 0.02 | 1.85 |
| rs372159963 | G/A | V279I | TOLERATED | 0.42 | 1.85 |
| rs759593783 | G/C | G275A | TOLERATED | 0.24 | 1.85 |
| rs775693036 | G/A | V270M | TOLERATED | 0.28 | 1.85 |
| rs764912508 | C/T | R264W | DAMAGING | 0.02 | 1.85 |
| rs752407216 | C/G | P261A | TOLERATED | 0.13 | 1.86 |
| rs143634072 | C/T | A253V | TOLERATED | 1 | 1.85 |
| rs369530077 | T/A | I251N | DAMAGING | 0.01 | 1.85 |
| rs369530077 | T/C | I251T | TOLERATED | 0.17 | 1.85 |
| rs757117798 | G/A | V242I | TOLERATED | 1 | 1.85 |
| rs774498455 | A/G | Q233R | TOLERATED | 0.39 | 1.85 |
| rs75992419 | T/C | S231P | TOLERATED | 0.1 | 1.85 |
| rs200326086 | C/T | L224F | DAMAGING | 0.01 | 1.85 |
| rs772408783 | C/G | L215V | DAMAGING | 0.02 | 1.85 |
| rs756988796 | C/T | R202W | TOLERATED | 0.08 | 1.85 |
| rs772165061 | G/C | V200L | TOLERATED | 0.23 | 1.85 |
| rs760115326 | T/G | F173L | TOLERATED | 0.1 | 1.85 |
| rs770463342 | G/T | K170N | TOLERATED | 0.09 | 1.85 |
| rs775116917 | C/T | A165V | TOLERATED | 0.23 | 1.92 |
| rs373089230 | G/A | M161I | TOLERATED | 0.47 | 1.84 |
| rs762544607 | T/C | M161T | TOLERATED | 0.45 | 1.84 |
| rs767286210 | C/T | L149F | TOLERATED | 0.32 | 1.85 |
| rs750288767 | G/A | V146M | TOLERATED | 0.12 | 1.85 |
| rs780018580 | T/C | F145L | TOLERATED | 0.75 | 1.85 |
| rs766267373 | T/C | L143P | DAMAGING | 0.01 | 1.85 |
| rs754925881 | C/T | R130C | TOLERATED | 0.17 | 1.85 |
| rs764226232 | C/T | R122W | TOLERATED | 0.17 | 1.88 |
| rs751626519 | T/A | M118K | TOLERATED | 0.2 | 1.84 |
| rs781585434 | C/T | P116S | TOLERATED | 0.18 | 1.85 |
| rs749789403 | G/A | D108N | DAMAGING | 0.02 | 1.85 |
| rs141511034 | C/T | P96L | TOLERATED | 0.33 | 1.85 |
| rs200676846 | G/A | R91H | TOLERATED | 0.09 | 1.93 |
| rs772885058 | G/C | E89D | TOLERATED | 0.31 | 1.95 |
| rs760402203 | G/A | V87I | TOLERATED | 0.29 | 1.99 |
| rs769729230 | G/A | E78K | TOLERATED | 0.15 | 2.04 |
| rs574996973 | C/T | P70S | TOLERATED | 0.75 | 2.23 |
| rs767693457 | G/A | E62K | TOLERATED | 0.26 | 2.25 |
| rs750597905 | G/T | C54F | TOLERATED | 0.15 | 2.35 |
| rs760949189 | T/C | C54R | TOLERATED | 0.54 | 2.35 |
| rs569050331 | G/C | E51Q | TOLERATED | 0.32 | 2.36 |
| rs769487573 | A/G | K47R | TOLERATED | 0.51 | 2.4 |
| rs762980942 | A/G | N44S | TOLERATED | 0.71 | 2.39 |
| rs773404208 | G/A | V41I | TOLERATED | 0.47 | 2.41 |
| rs369660216 | G/C | R40P | TOLERATED | 0.26 | 2.41 |
| rs766587890 | C/G | R40G | TOLERATED | 0.41 | 2.41 |
| rs754013380 | G/A | G38S | TOLERATED | 0.74 | 2.42 |
| rs751028180 | G/A | G31E | TOLERATED | 1 | 2.5 |
| rs149582032 | G/A | A28T | TOLERATED | 0.61 | 2.64 |
| rs780838176 | G/C | E26Q | TOLERATED | 0.34 | 2.72 |
| rs369139073 | C/T | T24M | TOLERATED | 0.12 | 2.81 |
| rs749145406 | G/C | A15P | TOLERATED | 0.35 | 2.92 |
| rs200868442 | G/A | G14F | TOLERATED | 0.91 | 2.92 |
| rs774465119 | T/G | N13K | TOLERATED | 0.93 | 2.92 |
| rs372656012 | G/A | G12E | TOLERATED | 1 | 3.01 |
| rs759643470 | T/A | V9E | TOLERATED | 0.74 | 3.01 |
| rs765487840 | A/T | H4L | DAMAGING *Warning! Low confidence | 0.03 | 3.36 |
| rs775780092 | C/T | H4Y | DAMAGING *Warning! Low confidence | 0.01 | 3.36 |

| **Table S2: List of non-synonymous SNPs of the human *ADIPOR1* gene analyzed by SNAP2 and nsSNPAnalyzer** | | | | | |
| --- | --- | --- | --- | --- | --- |
|  | | SNAP2 | | | nsSNPAnalyzer |
| rsIDs | Amino acid change | Prediction | Score | Expected accuracy | Prediction |
| rs764078304 | G367R | Effect | 72 | 85% | Disease |
| rs139371614 | G364S | Neutral | -74 | 87% | Disease |
| rs766580369 | Y363F | Neutral | -33 | 66% | Neutral |
| rs765425383 | A348G | Effect | 34 | 66% | Disease |
| rs778182434 | V342I | Neutral | -91 | 97% | Neutral |
| rs752071352 | H341Y | Effect | 80 | 91% | Disease |
| rs759555652 | R324L | Effect | 56 | 75% | Disease |
| rs780196089 | A319T | Neutral | -92 | 97% | Neutral |
| rs763241138 | I311V | Neutral | -22 | 61% | Neutral |
| rs12072660 | A307V | Neutral | -31 | 66% | Neutral |
| rs374623170 | T296R | Neutral | -43 | 72% | Neutral |
| rs757727437 | T295I | Neutral | -47 | 72% | Neutral |
| rs781601173 | K293R | Neutral | -49 | 72% | Neutral |
| rs749903174 | V292I | Neutral | -86 | 93% | Neutral |
| rs755615066 | A288T | Neutral | -95 | 97% | Neutral |
| rs779694619 | M283T | Neutral | -74 | 87% | Neutral |
| rs778848411 | V279G | Effect | 36 | 66% | Neutral |
| rs372159963 | V279I | Neutral | -87 | 93% | Neutral |
| rs759593783 | G275A | Effect | 45 | 71% | Disease |
| rs775693036 | V270M | Neutral | -63 | 82% | Neutral |
| rs764912508 | R264W | Effect | 49 | 71% | Disease |
| rs752407216 | P261A | Neutral | -22 | 61% | Neutral |
| rs143634072 | A253V | Neutral | -47 | 72% | Neutral |
| rs369530077 | I251N | Effect | 19 | 59% | Neutral |
| rs369530077 | I251T | Neutral | -65 | 82% | Neutral |
| rs757117798 | V242I | Neutral | -92 | 97% | Neutral |
| rs774498455 | Q233R | Neutral | -61 | 82% | Neutral |
| rs75992419 | S231P | Neutral | -49 | 72% | Neutral |
| rs200326086 | L224F | Effect | 2 | 53% | Disease |
| rs772408783 | L215V | Effect | 1 | 53% | Neutral |
| rs756988796 | R202W | Neutral | -35 | 66% | Disease |
| rs772165061 | V200L | Effect | 8 | 53% | Neutral |
| rs760115326 | F173L | Effect | 48 | 71% | Disease |
| rs770463342 | K170N | Effect | 25 | 63% | Neutral |
| rs775116917 | A165V | Neutral | -69 | 82% | Neutral |
| rs373089230 | M161I | Neutral | -46 | 72% | Neutral |
| rs762544607 | M161T | Neutral | -58 | 78% | Neutral |
| rs767286210 | L149F | Neutral | -50 | 72% | Disease |
| rs750288767 | V146M | Neutral | -40 | 66% | Neutral |
| rs780018580 | F145L | Neutral | -45 | 72% | Disease |
| rs766267373 | L143P | Effect | 75 | 85% | Disease |
| rs754925881 | R130C | Neutral | -28 | 61% | Neutral |
| rs764226232 | R122W | Neutral | -36 | 66% | Disease |
| rs751626519 | M118K | Effect | 60 | 80% | Disease |
| rs781585434 | P116S | Effect | 6 | 53% | Neutral |
| rs749789403 | D108N | Neutral | -10 | 53% | Neutral |
| rs141511034 | P96L | Neutral | -75 | 87% | Disease |
| rs200676846 | R91H | Neutral | -56 | 78% | Neutral |
| rs772885058 | E89D | Neutral | -86 | 93% | Neutral |
| rs760402203 | V87I | Neutral | -94 | 97% | Neutral |
| rs769729230 | E78K | Effect | 31 | 66% | Disease |
| rs574996973 | P70S | Neutral | -81 | 93% | Neutral |
| rs767693457 | E62K | Neutral | -30 | 61% | Neutral |
| rs750597905 | C54F | Neutral | -43 | 72% | Neutral |
| rs760949189 | C54R | Neutral | -52 | 78% | Neutral |
| rs569050331 | E51Q | Neutral | -72 | 87% | Neutral |
| rs769487573 | K47R | Neutral | -38 | 66% | Neutral |
| rs762980942 | N44S | Neutral | -71 | 87% | Neutral |
| rs773404208 | V41I | Neutral | -77 | 87% | Neutral |
| rs369660216 | R40P | Neutral | -8 | 53% | Neutral |
| rs766587890 | R40G | Neutral | -25 | 61% | Neutral |
| rs754013380 | G38S | Neutral | -69 | 82% | Neutral |
| rs751028180 | G31E | Neutral | -81 | 93% | Disease |
| rs149582032 | A28T | Neutral | -88 | 93% | Disease |
| rs780838176 | E26Q | Neutral | -89 | 93% | Disease |
| rs369139073 | T24M | Neutral | -60 | 78% | Neutral |
| rs749145406 | A15P | Neutral | -34 | 66% | Disease |
| rs200868442 | G14F | Neutral | -4 | 53% | Disease |
| rs774465119 | N13K | Effect | 18 | 59% | Disease |
| rs372656012 | G12E | Neutral | -63 | 82% | Disease |
| rs759643470 | V9E | Effect | 61 | 80% | Disease |
| rs765487840 | H4L | Neutral | -9 | 53% | Disease |
| rs775780092 | H4Y | Neutral | -27 | 61% | Disease |

| **Table S3: List of non-synonymous SNPs of the human *ADIPOR1* gene analyzed by PolyPhen-2** | | | | | |
| --- | --- | --- | --- | --- | --- |
| rsIDs | Amino acid change | PSID score | Sensitivity | Specificity | Prediction |
| rs764078304 | G367R | 0.150 | 0.89 | 0.71 | BENIGN |
| rs139371614 | G364S | 0.001 | 0.99 | 0.09 | BENIGN |
| rs766580369 | Y363F | 0.001 | 0.99 | 0.09 | BENIGN |
| rs765425383 | A348G | 0.992 | 0.49 | 0.95 | PROBABLY DAMAGING |
| rs778182434 | V342I | 0.100 | 0.91 | 0.68 | BENIGN |
| rs752071352 | H341Y | 0.997 | 0.27 | 0.98 | PROBABLY DAMAGING |
| rs759555652 | R324L | 0.946 | 0.65 | 0.92 | PROBABLY DAMAGING |
| rs780196089 | A319T | 0.028 | 0.94 | 0.59 | BENIGN |
| rs763241138 | I311V | 0.531 | 0.82 | 0.82 | POSSIBLY DAMAGING |
| rs12072660 | A307V | 0.250 | 0.87 | 0.75 | BENIGN |
| rs374623170 | T296R | 0.246 | 0.87 | 0.75 | BENIGN |
| rs757727437 | T295I | 0.017 | 0.95 | 0.54 | BENIGN |
| rs781601173 | K293R | 0.021 | 0.95 | 0.56 | BENIGN |
| rs749903174 | V292I | 0.012 | 0.96 | 0.52 | BENIGN |
| rs755615066 | A288T | 0.004 | 0.98 | 0.35 | BENIGN |
| rs779694619 | M283T | 0.026 | 0.94 | 0.58 | BENIGN |
| rs778848411 | V279G | 0.279 | 0.87 | 0.76 | BENIGN |
| rs372159963 | V279I | 0.055 | 0.93 | 0.64 | BENIGN |
| rs759593783 | G275A | 0.986 | 0.54 | 0.94 | PROBABLY DAMAGING |
| rs775693036 | V270M | 0.539 | 0.82 | 0.82 | POSSIBLY DAMAGING |
| rs764912508 | R264W | 0.665 | 0.79 | 0.84 | POSSIBLY DAMAGING |
| rs752407216 | P261A | 0.874 | 0.71 | 0.89 | POSSIBLY DAMAGING |
| rs143634072 | A253V | 0.679 | 0.79 | 0.85 | POSSIBLY DAMAGING |
| rs369530077 | I251N | 0.824 | 0.74; | 0.88 | POSSIBLY DAMAGING |
| rs369530077 | I251T | 0.034 | 0.94 | 0.60 | BENIGN |
| rs757117798 | V242I | 0.013 | 0.96 | 0.52 | BENIGN |
| rs774498455 | Q233R | 0.060 | 0.92 | 0.65 | BENIGN |
| rs75992419 | S231P | 0.625 | 0.80 | 0.83 | POSSIBLY DAMAGING |
| rs200326086 | L224F | 0.979 | 0.57 | 0.94 | PROBABLY DAMAGING |
| rs772408783 | L215V | 0.991 | 0.50 | 0.95 | PROBABLY DAMAGING |
| rs756988796 | R202W | 0.035 | 0.94 | 0.60 | BENIGN |
| rs772165061 | V200L | 0.775 | 0.76 | 0.86 | POSSIBLY DAMAGING |
| rs760115326 | F173L | 0.616 | 0.80 | 0.83 | POSSIBLY DAMAGING |
| rs770463342 | K170N | 0.965 | 0.62 | 0.93 | PROBABLY DAMAGING |
| rs775116917 | A165V | 0.097 | 0.93 | 0.85 | BENIGN |
| rs373089230 | M161I | 0.010 | 0.96 | 0.50 | BENIGN |
| rs762544607 | M161T | 0.010 | 0.96 | 0.50 | BENIGN |
| rs767286210 | L149F | 0.084 | 0.91 | 0.67 | BENIGN |
| rs750288767 | V146M | 0.531 | 0.82 | 0.82 | POSSIBLY DAMAGING |
| rs780018580 | F145L | 0.020 | 0.95 | 0.56 | BENIGN |
| rs766267373 | L143P | 0.996 | 0.36 | 0.97 | PROBABLY DAMAGING |
| rs754925881 | R130C | 0.260 | 0.87 | 0.75 | BENIGN |
| rs764226232 | R122W | 0.006 | 0.97 | 0.45 | BENIGN |
| rs751626519 | M118K | 0.391 | 0.84 | 0.79 | BENIGN |
| rs781585434 | P116S | 0.997 | 0.27 | 0.98 | PROBABLY DAMAGING |
| rs749789403 | D108N | 0.973 | 0.60 | 0.93 | PROBABLY DAMAGING |
| rs141511034 | P96L | 0.00 | 0.99 | 0.09 | BENIGN |
| rs200676846 | R91H | 0.003 | 0.98 | 0.26 | BENIGN |
| rs772885058 | E89D | 0.003 | 0.98 | 0.26 | BENIGN |
| rs760402203 | V87I | 0.026 | 0.94 | 0.58 | BENIGN |
| rs769729230 | E78K | 0.279 | 0.87 | 0.76 | BENIGN |
| rs574996973 | P70S | 0.992 | 0.49 | 0.95 | PROBABLY DAMAGING |
| rs767693457 | E62K | 0.734 | 0.85 | 0.92 | POSSIBLY DAMAGING |
| rs750597905 | C54F | 0.005 | 0.97; | 0.44 | BENIGN |
| rs760949189 | C54R | 0.006 | 0.97 | 0.45 | BENIGN |
| rs569050331 | E52Q | 0.006 | 0.97 | 0.45 | BENIGN |
| rs769487573 | K47R | 0.003 | 0.98 | 0.26 | BENIGN |
| rs762980942 | N44S | 0.000 | 1.00 | 0.000 | BENIGN |
| rs773404208 | V41I | 0.000 | 1.00 | 0.000 | BENIGN |
| rs369660216 | R40P | 0.000 | 1.00 | 0.000 | BENIGN |
| rs766587890 | R40G | 0.003 | 0.98 | 0.26 | BENIGN |
| rs754013380 | G38S | 0.038 | 0.93 | 0.61 | BENIGN |
| rs751028180 | G31E | 0.960 | 0.63 | 0.92 | PROBABLY DAMAGING |
| rs149582032 | A28T | 0.000 | 1.00 | 0.000 | BENIGN |
| rs780838176 | E26Q | 0.813 | 0.75; | 0.87 | POSSIBLY DAMAGING |
| rs369139073 | T24M | 0.057 | 0.93 | 0.64 | BENIGN |
| rs749145406 | A15P | 0.000 | 1.00 | 0.000 | BENIGN |
| rs200868442 | G14F | 0.163 | 0.89 | 0.72 | BENIGN |
| rs774465119 | N13K | 0.000 | 1.00 | 0.000 | BENIGN |
| rs372656012 | G12E | 0.001 | 0.99 | 0.99 | BENIGN |
| rs759643470 | V9E | 0.016 | 0.95 | 0.79 | BENIGN |
| rs765487840 | H4L | 0.002 | 0.99 | 0.18 | BENIGN |
| rs775780092 | H4Y | 0.018 | 0.95 | 0.55 | BENIGN |

| **Table S4: List of non-synonymous SNPs of the human *ADIPOR1* gene analyzed by PhD-SNP, PANTHER, and SNPs&GO** | | | | | | | | | |
| --- | --- | --- | --- | --- | --- | --- | --- | --- | --- |
|  | PhD-SNP | | | PANTHER | | | SNPs&GO | | |
| A A change | Prediction | RI | Probability | Prediction | RI | Probability | Prediction | RI | Probability |
| G367R | Disease | 2 | 0.602 | Neutral | 4 | 0.276 | Neutral | 6 | 0.208 |
| G364S | Neutral | 7 | 0.151 | Neutral | 8 | 0.096 | Neutral | 9 | 0.050 |
| Y363F | Neutral | 5 | 0.228 | Neutral | 8 | 0.096 | Neutral | 9 | 0.044 |
| A348G | Disease | 8 | 0.897 | Disease | 6 | 0.792 | Disease | 6 | 0.795 |
| V342I | Neutral | 3 | 0.327 | Neutral | 8 | 0.090 | Neutral | 8 | 0.082 |
| H341Y | Disease | 9 | 0.963 | Disease | 9 | 0.961 | Disease | 8 | 0.904 |
| R324L | Disease | 9 | 0.951 | Disease | 8 | 0.908 | Disease | 8 | 0.909 |
| A319T | Neutral | 7 | 0.171 | Neutral | 8 | 0.122 | Neutral | 8 | 0.046 |
| I311V | Neutral | 0 | 0.485 | Neutral | 6 | 0.200 | Neutral | 7 | 0.143 |
| A307V | Disease | 2 | 0.615 | Neutral | 0 | 0.489 | Disease | 1 | 0.528 |
| T296R | Disease | 2 | 0.599 | Neutral | 5 | 0.269 | Neutral | 3 | 0.340 |
| T295I | Neutral | 3 | 0.334 | Neutral | 8 | 0.095 | Neutral | 8 | 0.081 |
| K293R | Neutral | 5 | 0.231 | Neutral | 6 | 0.185 | Neutral | 8 | 0.115 |
| V292I | Neutral | 9 | 0.072 | Neutral | 7 | 0.150 | Neutral | 9 | 0.030 |
| A288T | Neutral | 8 | 0.106 | Neutral | 7 | 0.144 | Neutral | 9 | 0.065 |
| M283T | Neutral | 4 | 0.313 | Neutral | 4 | 0.291 | Neutral | 4 | 0.279 |
| V279G | Disease | 5 | 0.737 | Disease | 6 | 0.777 | Disease | 2 | 0.617 |
| V279I | Neutral | 7 | 0.171 | Neutral | 8 | 0.108 | Neutral | 9 | 0.039 |
| G275A | Neutral | 0 | 0.477 | Disease | 8 | 0.915 | Disease | 2 | 0.600 |
| V270M | Neutral | 7 | 0.153 | Disease | 3 | 0.661 | Neutral | 6 | 0.199 |
| R264W | Disease | 8 | 0.896 | Disease | 10 | 0.985 | Disease | 7 | 0.827 |
| P261A | Neutral | 4 | 0.319 | Neutral | 4 | 0.320 | Neutral | 7 | 0.133 |
| A253V | Neutral | 3 | 0.374 | Neutral | 3 | 0.351 | Neutral | 5 | 0.259 |
| I251N | Disease | 6 | 0.801 | Disease | 5 | 0.737 | Disease | 6 | 0.819 |
| I251T | Neutral | 0 | 0.486 | Neutral | 3 | 0.345 | Neutral | 4 | 0.312 |
| V242I | Neutral | 9 | 0.068 | Neutral | 8 | 0.096 | Neutral | 10 | 0.022 |
| Q233R | Neutral | 2 | 0.390 | Neutral | 5 | 0.260 | Neutral | 7 | 0.174 |
| S231P | Disease | 6 | 0.807 | Neutral | 3 | 0.332 | Neutral | 0 | 0.493 |
| L224F | Disease | 3 | 0.634 | Neutral | 1 | 0.461 | Neutral | 4 | 0.319 |
| L215V | Disease | 3 | 0.661 | Neutral | 1 | 0.453 | Neutral | 2 | 0.378 |
| R202W | Disease | 3 | 0.670 | Disease | 7 | 0.852 | Disease | 1 | 0.554 |
| V200L | Neutral | 3 | 0.343 | Neutral | 6 | 0.204 | Neutral | 8 | 0.116 |
| F173L | Disease | 2 | 0.596 | Neutral | 5 | 0.227 | Neutral | 5 | 0.268 |
| K170N | Neutral | 3 | 0.361 | Disease | 3 | 0.636 | Neutral | 3 | 0.366 |
| A165V | Neutral | 0 | 0.487 | Neutral | 7 | 0.129 | Neutral | 8 | 0.099 |
| M161I | Neutral | 6 | 0.208 | Neutral | 7 | 0.146 | Neutral | 9 | 0.059 |
| M161T | Neutral | 7 | 0.169 | Neutral | 9 | 0.072 | Neutral | 9 | 0.043 |
| L149F | Neutral | 4 | 0.296 | Neutral | 7 | 0.163 | Neutral | 9 | 0.040 |
| V146M | Neutral | 2 | \| 0.418 \|  \|  \| \| --- \| --- \| --- \| | \| Neutral \|  \| \| --- \| --- \| | 1 | \| 0.531 \|  \|  \| \| --- \| --- \| --- \| | Neutral | 1 | \| 0.294 \|  \|  \| \| --- \| --- \| --- \| |
| F145L | \| Disease \|  \| \| --- \| --- \| | 1 | \| 0.536 \|  \|  \| \| --- \| --- \| --- \| | \| Neutral \|  \| \| --- \| --- \| | 8 | \| 0.080 \|  \|  \| \| --- \| --- \| --- \| | \| Neutral \|  \| \| --- \| --- \| | 6 | 0.177 |
| L143P | Disease | 9 | \| 0.942 \|  \|  \| \| --- \| --- \| --- \| | Disease | 4 | \| 0.719 \|  \|  \| \| --- \| --- \| --- \| | Disease | 7 | \| 0.837 \|  \|  \| \| --- \| --- \| --- \| |
| R130C | \| Disease \|  \| \| --- \| --- \| | 4 | \| 0.715 \|  \|  \| \| --- \| --- \| --- \| | \| Disease \|  \| \| --- \| --- \| | 4 | \| 0.682 \|  \|  \| \| --- \| --- \| --- \| | \| Disease \|  \| \| --- \| --- \| | 1 | 0.542 |
| R122W | Disease | 2 | \| 0.608 \|  \|  \| \| --- \| --- \| --- \| | Disease | 7 | 0.827 | \| Neutral \|  \| \| --- \| --- \| | 3 | \| 0.370 \|  \|  \| \| --- \| --- \| --- \| |
| M118K | \| Disease \|  \| \| --- \| --- \| | 1 | \| 0.538 \|  \|  \| \| --- \| --- \| --- \| | \| Disease \|  \| \| --- \| --- \| | 3 | \| 0.626 \|  \|  \| \| --- \| --- \| --- \| | \| Disease \|  \| \| --- \| --- \| | 1 | \| 0.545 \|  \|  \| \| --- \| --- \| --- \| |
| P116S | Neutral | 1 | 0.473 | Disease | 4 | 0.697 | Disease | 0 | 0.509 |
| D108N | Disease | 0 | 0.523 | Neutral | 3 | 0.339 | Neutral | 2 | 0.394 |
| P96L | Neutral | 1 | 0.431 | Neutral | 4 | 0.279 | Neutral | 5 | 0.232 |
| R91H | Disease | 5 | 0.743 | Neutral | 3 | 0.366 | Neutral | 1 | 0.455 |
| E89D | Disease | 3 | 0.666 | Neutral | 1 | 0.445 | Disease | 1 | 0.534 |
| V87I | Neutral | 5 | 0.252 | Neutral | 7 | 0.156 | Neutral | 9 | 0.070 |
| E78K | Disease | 5 | 0.742 | Neutral | 3 | 0.332 | Neutral | 2 | 0.414 |
| P70S | Neutral | 8 | 0.11 | Neutral | 8 | 0.093 | Neutral | 9 | 0.035 |
| E62K | Neutral | 5 | 0.235 | Neutral | 7 | 0.161 | Neutral | 8 | 0.080 |
| C54F | Neutral | 6 | 0.178 | Neutral | 9 | 0.060 | Neutral | 10 | 0.023 |
| C54R | Neutral | 6 | 0.215 | Neutral | 9 | 0.060 | Neutral | 9 | 0.039 |
| E51Q | Neutral | 8 | 0.120 | Neutral | 6 | 0.208 | Neutral | 9 | 0.039 |
| K47R | Neutral | 8 | 0.083 | Unclassified | NA | NA | Neutral | 10 | 0.024 |
| N44S | Neutral | 9 | 0.059 | Unclassified | NA | NA | Neutral | 10 | 0.015 |
| V41I | Neutral | 9 | 0.027 | Unclassified | NA | NA | Neutral | 10 | 0.008 |
| R40P | Disease | 2 | 0.622 | Unclassified | NA | NA | Neutral | 6 | 0.187 |
| R40G | Neutral | 5 | 0.267 | Unclassified | NA | NA | Neutral | 8 | 0.081 |
| G38S | Neutral | 6 | 0.224 | Unclassified | NA | NA | Neutral | 9 | 0.038 |
| G31E | Neutral | 4 | 0.284 | Unclassified | NA | NA | Neutral | 8 | 0.079 |
| A28T | Neutral | 8 | 0.113 | Unclassified | NA | NA | Neutral | 9 | 0.032 |
| E26Q | Neutral | 6 | 0.216 | Unclassified | NA | NA | Neutral | 9 | 0.040 |
| T24M | Neutral | 5 | 0.273 | Unclassified | NA | NA | Neutral | 9 | 0.068 |
| A15P | Disease | 3 | 0.626 | Unclassified | NA | NA | Neutral | 6 | 0.186 |
| G14F | Disease | 2 | 0.615 | Unclassified | NA | NA | Neutral | 8 | 0.119 |
| N13K | Disease | 1 | 0.543 | Unclassified | NA | NA | Neutral | 7 | 0.146 |
| G12E | Disease | 0 | 0.521 | Unclassified | NA | NA | Neutral | 7 | 0.148 |
| V9E | Disease | 3 | 0.635 | Unclassified | NA | NA | Neutral | 6 | 0.190 |
| H4L | Neutral | 3 | 0.332 | Unclassified | NA | NA | Neutral | 9 | 0.067 |
| H4Y | Neutral | 3 | 0.351 | Unclassified | NA | NA | Neutral | 8 | 0.080 |

| **Table S5: List of non-synonymous SNPs of the human *ADIPOR1* gene analyzed by Fathmm** | | |
| --- | --- | --- |
| Amino acid change | Prediction | Score |
| G367R | TOLERATED | 1.81 |
| G364S | TOLERATED | 2.01 |
| Y363F | TOLERATED | 1.91 |
| A348G | TOLERATED | 1.17 |
| V342I | TOLERATED | 1.36 |
| H341Y | TOLERATED | -0.3 |
| R324L | TOLERATED | 0.82 |
| A319T | TOLERATED | 1.63 |
| I311V | TOLERATED | 1.5 |
| A307V | TOLERATED | 1.63 |
| T296R | TOLERATED | 1.93 |
| T295I | TOLERATED | 1.57 |
| K293R | TOLERATED | 1.53 |
| V292I | TOLERATED | 1.54 |
| A288T | TOLERATED | 1.6 |
| M283T | TOLERATED | 1.67 |
| V279G | TOLERATED | 1.2 |
| V279I | TOLERATED | 1.44 |
| G275A | TOLERATED | 1.13 |
| V270M | TOLERATED | 1.46 |
| R264W | TOLERATED | 1.19 |
| P261A | TOLERATED | 1.58 |
| A253V | TOLERATED | 1.54 |
| I251N | TOLERATED | 1.38 |
| I251T | TOLERATED | 1.42 |
| V242I | TOLERATED | 1.77 |
| Q233R | TOLERATED | 1.58 |
| S231P | TOLERATED | 1.52 |
| L224F | TOLERATED | 1.38 |
| L215V | TOLERATED | 1.17 |
| R202W | TOLERATED | 1.52 |
| V200L | TOLERATED | 1.56 |
| F173L | TOLERATED | 1.5 |
| K170N | TOLERATED | 1.47 |
| A165V | TOLERATED | 1.84 |
| M161I | TOLERATED | 1.61 |
| M161T | TOLERATED | 1.59 |
| L149F | TOLERATED | 1.37 |
| V146M | TOLERATED | 1.47 |
| F145L | TOLERATED | 1.58 |
| L143P | TOLERATED | 1.24 |
| R130C | TOLERATED | 1.51 |
| R122W | DAMAGING | -4.2 |
| M118K | DAMAGING | -4.01 |
| P116S | DAMAGING | -3.97 |
| D108N | DAMAGING | -4.1 |
| P96L | DAMAGING | -4.03 |
| R91H | DAMAGING | -4.18 |
| E89D | DAMAGING | -4.12 |
| V87I | DAMAGING | -4.12 |
| E78K | DAMAGING | -4.08 |
| P70S | DAMAGING | -4.01 |
| E62K | DAMAGING | -4.09 |
| C54F | DAMAGING | -4.06 |
| C54R | DAMAGING | -3.99 |
| E51Q | DAMAGING | -4.08 |
| K47R | DAMAGING | -4.14 |
| N44S | DAMAGING | -3.97 |
| V41I | DAMAGING | -4.09 |
| R40P | DAMAGING | -4.14 |
| R40G | DAMAGING | -4.11 |
| G38S | DAMAGING | -3.99 |
| G31E | DAMAGING | -3.99 |
| A28T | DAMAGING | -4.08 |
| E26Q | DAMAGING | -4.15 |
| T24M | DAMAGING | -4.12 |
| A15P | DAMAGING | -4.17 |
| G14F | DAMAGING | -4.1 |
| N13K | DAMAGING | -4.02 |
| G12E | DAMAGING | -3.98 |
| V9E | DAMAGING | -3.98 |
| H4L | DAMAGING | -4.05 |
| H4Y | DAMAGING | -4.08 |
